# Circulating Erythrocyte‐ and Endothelial‐Derived Microvesicles as Biomarkers of Hemolysis and Clinical Severity in Sickle Cell Disease

**DOI:** 10.1111/jcmm.70827

**Published:** 2025-10-13

**Authors:** Khouloud Khalfaoui, Mariem Chebbi, Oussema Souiai, Ilhem Ben Fraj, Monia Ben Khaled, Amine Hableni, Mbarka Barmate, Dorra Chaouachi, Fethi Mellouli, Monia Ouederni, Ines Safra, Samia Menif, Imen Moumni

**Affiliations:** ^1^ Laboratory of Molecular and Cellular Hematology (LMCH) Institut Pasteur de Tunis Tunis Tunisia; ^2^ Laboratory of Bioinformatics, Biomathematics and Biostatistics (BIMS) Institut Pasteur de Tunis Tunis Tunisia; ^3^ Higher Institute of Biotechnology of Béja University of Jendouba Jendouba Tunisia; ^4^ Department of Pediatrics: Immunology, Hematology, and Stem Cell Transplantation National Bone Marrow Transplant Center Tunis Tunisia

**Keywords:** circulating microvesicles, fetal hemoglobin, flow cytometry, LDH, sickle cell disease

## Abstract

Sickle cell disease (SCD) is a monogenic disorder caused by the presence of hemoglobin S (Hb S) and is associated with a wide range of clinical complications, including hemolytic anemia, vaso‐occlusive crises (VOC), and acute chest syndrome (ACS). This variability is largely driven by Hb S polymerisation and abnormal cell adhesion, which promote the release of circulating microvesicles (MVs). MVs are small vesicles (0.1–1.0 μm) released from various cell types in response to oxidative stress, cellular activation, or apoptosis. They possess pro‐coagulant and pro‐inflammatory properties and are increasingly recognised as potential modulators of disease processes. In this study, homozygous SCD patients and healthy controls were recruited to characterise their MVs profiles using flow cytometry and to explore associations with clinical and biological parameters. SCD patients exhibited significantly elevated levels of MVs compared to controls. Notably, red cell–derived MVs (RMVs) and phosphatidylserine‐positive MVs (AMVs) were strongly associated with elevated lactate dehydrogenase (LDH) levels and clinical severity. A negative correlation was observed between fetal hemoglobin (Hb F) and endothelial MVs (EMVs), suggesting a protective role of Hb F against endothelial injury. These findings support the potential of MVs as diagnostic and prognostic biomarkers for identifying SCD patients at higher risk of complications.

## 1 Introduction

Sickle cell disease (SCD) is an autosomal recessive disorder caused by a point mutation in the *HBB* gene, resulting in the substitution of valine for glutamic acid at the sixth position of the β‐globin chain [1]. This mutation produces abnormal hemoglobin S (HbS) that polymerises under hypoxic conditions, deforming erythrocytes into a sickle shape. These rigid cells obstruct small blood vessels, causing microvascular occlusion and a cascade of clinical complications [2].

In Tunisia, the prevalence of SCD reaches 1.89%, making it one of the most frequent hemoglobinopathies [3]. The disease manifests with a wide spectrum of complications. One of the most common manifestations of the disease is hemolytic anemia, which is caused by the premature destruction of sickled red blood cells, resulting in fatigue, jaundice, and other long‐term complications [2]. Acute complications include vaso‐occlusive crises (VOCs), the hallmark of SCD, characterised by sudden, intense pain and tissue ischaemia [4, 5]. Acute chest syndrome (ACS), a leading cause of mortality, presents with hypoxia and pulmonary infiltrates [6]. Neurological events such as overt strokes and silent cerebral infarctions contribute to long‐term cognitive impairments [7]. Other complications include priapism, hepatic and splenic sequestration, where blood pools in these organs, causing enlargement and potential damage [2, 8, 9]. These manifestations necessitate comprehensive and multidisciplinary management.

The complex pathophysiology of SCD is not limited to the presence of sickled erythrocytes but also involves a chronic inflammatory state, endothelial dysfunction, and a hypercoagulable environment [10]. In recent years, increasing attention has been directed toward circulating microvesicles (MVs), submicron extracellular vesicles with a diameter ranging from 100 to 1000 nm, released from various cell types, including erythrocytes, endothelial cells, platelets, and leukocytes [11, 12, 13]. MVs are generated during cellular activation, apoptosis, or in response to stressors such as hypoxia, oxidative stress, and inflammation [14, 15]. Their biogenesis involves calcium influx and the activation of scramblases and floppases, which translocate phosphatidylserine (PS) to the outer membrane leaflet, facilitating vesicle budding [16, 17, 18]. Simultaneously, changes in membrane lipid composition and clustering of transmembrane proteins are promoted [19].

These MVs, heterogeneous in origin and composition, carry phospholipids, surface antigens, and membrane proteins, including PS, a key procoagulant component that supports thrombin generation and contributes to hypercoagulability in various disease states [20, 21]. Platelet‐derived MVs (PMVs), the most abundant subtype, express P‐selectin, CD41, CD61, and platelet‐endothelial adhesion molecules, and are central to thrombosis and haemostasis [15, 17, 22, 23]. Endothelial‐derived MVs (EMVs) express adhesion molecules such as ICAM‐1, E‐selectin, and cadherins, and contribute to inflammation and angiogenesis [12, 24]. Red cell‐derived MVs (RMVs) express CD235a and CD47; CD47 confers immune evasion, while CD235a serves as a general erythrocyte marker. MVs can also bind anticoagulant proteins, such as protein S [25, 26]. MVs and transfer proteins, lipids, and nucleic acids to recipient cells, modulating diverse biological responses [20].

Elevated levels of MVs have been associated with disease progression in cardiovascular, cancer, and autoimmune diseases, where they are implicated in endothelial damage, inflammation and thrombosis [27, 28, 29]. In SCD, MVs have been identified as important mediators of vascular complications. Their increased levels correlate with VOC frequency, endothelial activation, and enhanced erythrocyte adhesion, amplifying inflammation and vaso‐occlusion [12, 30].

In this study, we investigate the role of MVs in the complications associated with SCD. We analyze the quantitative and qualitative profiles of MVs in SCD patients and compare them to those of healthy donors. Furthermore, we examine the associations between MVs profiles and the clinical manifestations among SCD patients to identify potential biomarkers for diagnosis or therapeutic targeting.

## 2 Materials and Methods

### 2.1 Patients and Blood Sampling

Sixty‐eight individuals with homozygous SCD and sixty‐two healthy donors were included in our study. SCD patients were recruited from the Department of Pediatrics at the Bone Marrow Transplant Center of Tunisia. Upon recruitment, all patients were stable, without vaso‐occlusive crises or other acute medical complications, and had not recently received blood transfusions. Healthy donors were identified at the Pasteur Institute of Tunisia. They had no history of chronic or acute inflammatory or vascular diseases. A complete blood count and haemoglobin electrophoresis were performed to confirm the absence of hereditary haemoglobin abnormalities. The National Ethics Committee of Tunisia approved the study under the reference 2018/11/I/LR16IPT07/V1. Written informed consent was obtained from all participants.

### 2.2 Purification of Plasmatic Microvesicles

Peripheral blood was collected by venipuncture using an appropriately sized needle to minimise haemolysis, and the initial few millilitres of blood were discarded to prevent contamination from the first puncture. Peripheral blood was then collected into citrate‐containing tubes. During transport, samples were kept in an upright position to minimise mechanical stress and prevent artificial activation of cells or the release of MVs. Sample processing began within two hours of collection, as delays beyond this point may change MVs concentration and composition due to ongoing cellular activation or degradation. Within this two‐hour interval, samples underwent two centrifugation steps at 1500×*g* for 15 min at 20°C to separate plasma from erythrocytes and leukocytes. The resulting supernatant, designated as platelet‐poor plasma (PPP), was carefully transferred to new tubes. A third centrifugation at 13,000×*g* for 2 min was performed to remove residual platelets, yielding platelet‐free plasma (PFP). Aliquots of 200 μL of PFP were prepared and stored at −80°C until further analysis.

### 2.3 Characterization of Microvesicles

MVs released from various cell types expose PS on their outer membrane, a marker typically confined to the inner leaflet in intact cells. Flow cytometry was used to analyse both the size distribution and cellular origin of MVs. Megamix beads (BioCytex) were used as calibration beads to standardise size gates and ensure reproducible measurements across samples and Perfect‐Count microspheres (Cytognos, Spain) were used to determine absolute MVs concentrations based on the manufacturer's formula.

To identify the cellular origin of MVs, 38 μL of PFP was incubated for 30 min at room temperature in the dark with 5 μL of each of the following fluorochrome‐conjugated antibodies: Annexin V‐FITC (BD Pharmingen), anti‐CD235a‐PE‐Cy7 (EXBIO Praha, Czech Republic), anti‐CD41‐APC (EXBIO Praha, Czech Republic), and anti‐CD144‐PE (EXBIO Praha, Czech Republic). These antibodies were selected to identify PS positive MVs with (Annexin V), derived from erythrocytes (CD235a), platelets (CD41), and endothelial cells (CD144). Dual staining with Annexin V and each cell‐specific marker allowed identification of PS‐positive MVs from specific cellular origins.

Following incubation, diluted Annexin V Binding Buffer (BD Pharmingen) and an appropriate volume of Perfect‐Count microspheres (Cytognos, Spain) were added according to the manufacturer's instructions. As a control, 500 μL of Megamix beads was added to a separate control tube to validate gating. Samples were analysed using a BD FACSCanto II flow cytometer equipped with BD FACSDiva software, and MVs were identified based on forward scatter (FSC), side scatter (SSC), and Annexin V binding. A total of 10,000 events were collected per sample. Fluorescence from FITC and PE/APC fluorochromes was excited by 488‐ and 633‐nm lasers, respectively, and detected using appropriate photomultipliers. The absolute MVs concentration (MVs/μL) was calculated using the following formula:

$$\begin{array}{c} \text{Absolute MVs concentration}\;\left(\mathrm{MVs}/\text{μL}\right)=\\ \;\left(\left[\text{Number of MVs events}\right]/\left[\text{Volume of}\;\mathrm{PFP}\times \text{Total number of events}\right]\right)\\ \;\times \left(\text{Number of Perfect Count beads}/\text{μL}\right). \end{array}$$

These procedures, including the use of Megamix for standardization and Perfect‐Count beads for absolute counts, ensured reproducible and reliable quantification of MVs across all samples.

### 2.4 Clinical Evaluation

A clinical assessment was performed for each patient to evaluate their health status at the time of blood sampling and during the preceding period. This evaluation included the documentation of major sickle cell‐related complications, namely VOC, stroke, splenic sequestration, and ACS, as well as the use of hydroxyurea (HU) treatment. Each complication was marked as either present or absent, and a specific score was assigned to each complication based on its clinical severity. A total clinical severity score was then calculated for each patient by adding the scores of all present complications. This approach allowed for the stratification of patients into clinical subgroups based on disease severity and treatment status. The findings from this assessment contributed to a better understanding of the role of MVs in the pathophysiology of SCD and offered insights into patient‐specific responses to HU therapy.

### 2.5 Statistical Methods

Data analysis was performed using Python. The dataset was loaded with the *pandas* library, and all plots were generated using *matplotlib* and *seaborn*. Shapiro normality tests were performed for all experimental variables, indicating that the data were not normally distributed. Prior to group comparisons, normality tests were performed to validate the use of the Mann–Whitney *U* test. Statistical significance was defined as *p* < 0.05. Spearman correlation analysis was performed to assess the relationship between experimental variables and the clinical and biological parameters of the patients. Correlation results between numerical variables were visualised in a heatmap.

## 3 Results

### 3.1 Hematological and Biochemical Characteristics of the Study Population

Our study population included 62 healthy donors and 68 patients with homozygous SCD. The patient group was subdivided into 42 individuals receiving hydroxyurea treatment and 26 untreated individuals. Treated patients had been on hydroxyurea therapy for a minimum of three months, whereas untreated patients had not initiated this treatment at the time of sampling. The sex ratio was balanced across all groups. Haematological parameters, including haemoglobin concentration, fetal haemoglobin (HbF), leukocyte count and platelet count, were assessed in all participants. In addition, neutrophil counts and lactate dehydrogenase (LDH) levels, considered markers of inflammation and haemolysis, were evaluated exclusively in the SCD groups. Compared to healthy controls, SCD patients present significantly lower levels of Hb and elevated leukocyte and platelet counts (*p* < 0.0001). These findings are consistent with the typical haematological profile of SCD. Table 1 summarizes the haematological and biochemical findings, with *p*‐values considered statistically significant at *p* < 0.05. Data are expressed as median values with interquartile ranges (IQR).

**TABLE 1:** Hematological and biochemical parameters in SCD patients and controls.

Parameters	SCD patients (*n* = 68)	Controls (*n* = 62)	*p*
Sex ratio (M^a^/F^b^)	0.94 (33/35)	0.68 (25/37)	—
Age (years)	11.0 [7.0–15.5]	28.0 [24.0–32.0]	—
Fetal hemoglobin (%)	16.3 [10.2–23.9]	0.0 [0.0–0.0]	< 0.0001
Hemoglobin (g/dL)^c^	8.8 [7.6–9.5]	13.5 [12.7–14.2]	< 0.0001
Leukocytes (×10^c^/μL)^d^	10.5 [8.6–13.1]	6.4 [5.6–6.9]	< 0.0001
Neutrophils (×10^c^/μL)	5.0 [3.8–6.6]	—	—
Platelets (×10^c^/μL)	407.0 [290.3–487.5]	226.0 [186.3–249.8]	< 0.0001
LDH^e^ (IU/L)^f^	500.0 [418.5–663.5]	—	—

*Note:* Data are expressed as median values with interquartile ranges [IQR]. Compared to controls, SCD patients exhibit significantly lower haemoglobin levels and elevated leukocyte and platelet counts (*p* < 0.0001 for all). These findings are consistent with the typical haematological profile of SCD.

^a^ Male.

^b^ Female.

^c^ Grams per decilitre.

^d^ Thousands of cells per microlitre.

^e^ Lactate dehydrogenase.

^f^ International units per litre.

### 3.2 MVs Profiles in Healthy Subjects and SCD Patients

**FIGURE 1:**
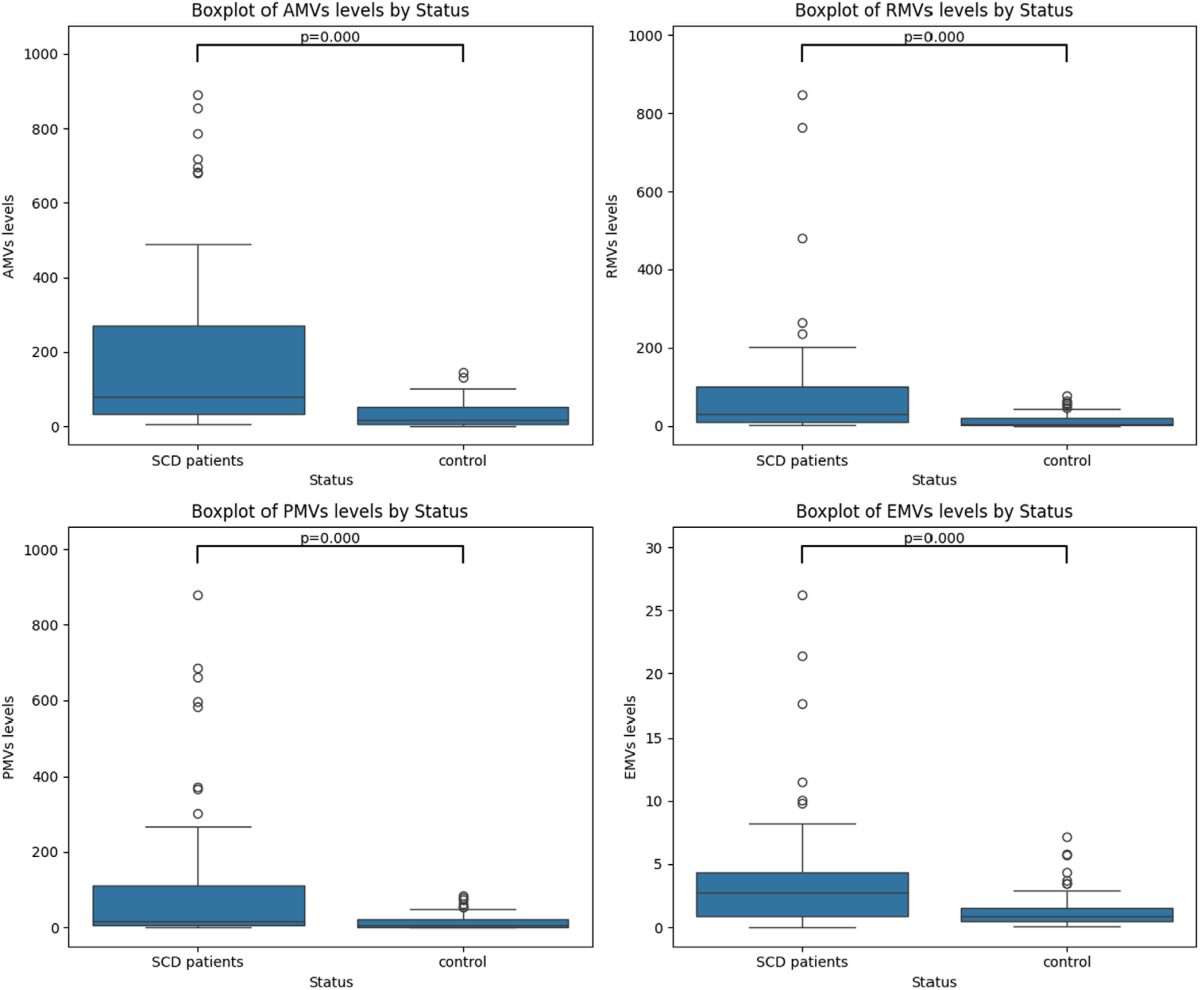
Comparison of MVs subtypes between SCD patients and healthy controls. Boxplots comparing the levels of MVs subtypes between SCD patients and healthy controls. Significantly higher levels of all MV subtypes were observed in SCD patients, including phosphatidylserine‐positive MVs (AMVs; *p* < 0.0001), erythrocyte‐derived MVs (RMVs; *p* < 0.0001), platelet‐derived MVs (PMVs; *p* < 0.0001), and endothelial‐derived MVs (EMVs; *p* < 0.0001). These findings indicate elevated levels of circulating MVs in SCD, reflecting increased cellular stress, haemolysis, and endothelial dysfunction, key features of the disease's pathophysiology.

The levels of different MVs subtypes, phosphatidylserine‐positive MVs (AMVs), erythrocyte‐derived (RMVs), platelet‐derived (PMVs), and endothelial‐derived (EMVs), were compared between healthy donors and patients with SCD using the Mann–Whitney U test. SCD patients showed significantly elevated levels of all MVs subtypes compared to healthy controls, with *p* < 0.0001. These results reflect increased cellular stress, apoptosis, and endothelial dysfunction. Figure 1 illustrates the ranked distributions of AMVs, RMVs, PMVs, and EMVs across the two study groups. Table 2 presents the median with interquartile range (IQR) for each MV subtype.

**TABLE 2:** MVs subtypes in SCD patients and healthy controls: median [IQR] values.

MVs subtypes	SCD patients median [IQR] (MVs/μL)	Controls median [IQR] (MVs/μl)	*p*
AMVs^a^	78.66 [32.06–270.47]	18.03 [7.19–53.62]	< 0.0001
RMVs^b^	31.45 [10.01–99.73]	4.88 [2.19–19.20]	< 0.0001
PMVs^c^	16.31 [5.50–110.67]	6.03 [0.71–21.65]	< 0.0001
EMVs^d^	2.74 [0.92–4.33]	0.87 [0.51–1.54]	< 0.0001

*Note:* presents a detailed comparison of circulating MVs subtypes between SCD patients and healthy controls. Across all MVs subtypes, AMVs, RMVs, PMVs, and EMVs, SCD patients showed significantly elevated levels compared to controls, with *p* < 0.0001 for each comparison. These findings confirm a marked increase in cell‐derived MVs in the pathological state of SCD, reflecting enhanced cell activation, apoptosis, and endothelial dysfunction.

^a^ Phosphatidylserine‐positive microvesicles.

^b^ Erythrocyte‐derived microvesicles.

^c^ Platelet‐derived microvesicles.

^d^ Endothelial‐derived microvesicles.

**FIGURE 2:**
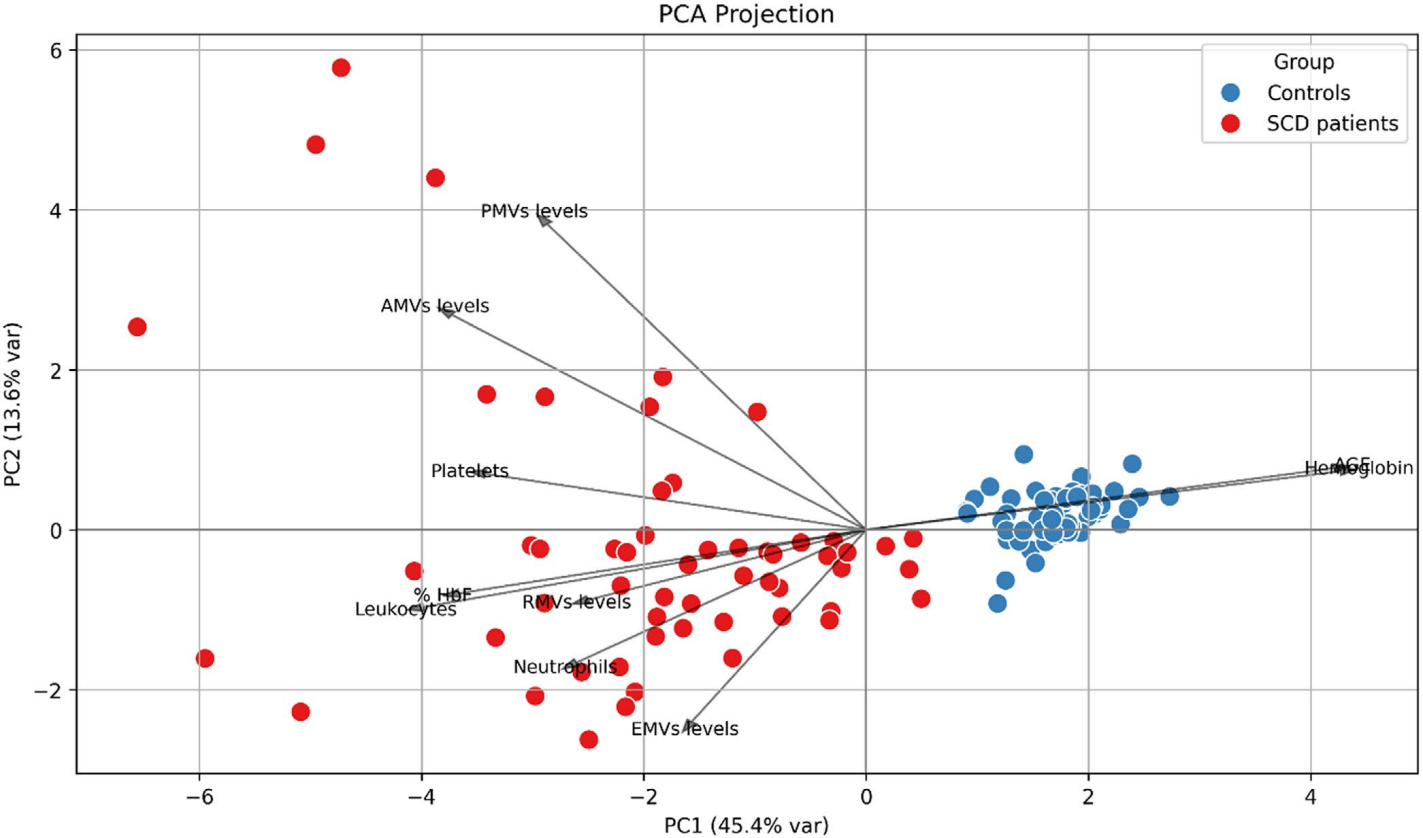
Principal component analysis (PCA) distinguishing SCD patients from healthy controls according to plasmatic microvesicles levels and hemoglobin parameters. The PCA plot reveals a clear separation between healthy controls (blue) and SCD patients (red) along PC1, which explains 44.6% of the variance. This clustering reflects distinct differences in hemoglobin levels and MVs burden. SCD patients are characterised by elevated RMVs and PMVs, as well as increased platelet counts, while controls cluster around higher hemoglobin.

Principal Component Analysis (PCA) separates healthy controls from SCD patients based on MVs burden and hemoglobin profiles. Healthy controls present normal haematological parameters and low levels of MVs, with no signs of haemolysis or vascular damage. In contrast, SCD patients show elevated levels of MVs (particularly RMVs and PMVs), increased platelet counts, and decreased levels of total haemoglobin, confirming the presence of anaemia. The Spearman test revealed a moderate negative correlation between Hb and AMVs (*r* = −0.31) and RMVs (*r* = −0.35), indicating that higher levels of AMVs and RMVs are associated with lower haemoglobin. These findings suggest elevated MVs, especially RMVs and PMVs, may contribute to ongoing haemolytic anaemia and vascular stress in SCD. Figure 2 summarises the main findings of the PCA.

### 3.3 Influence of Sex and Age on MVs Levels

Our preliminary results indicate that sex does not significantly influence the variability of MVs levels. According to the Mann–Whitney test, there were no statistically significant differences in the levels of AMVs, RMVs, PMVs, and EMVs between males and females, with *p*‐values of 0.075, 0.291, 0.123 and 0.688, respectively (Table 3). To assess age‐related differences, patients were divided into two groups: G1 (< 12 years; *n* = 41) and G2 (> 12 years; *n* = 27). Again, no significant differences were observed between the groups, with *p*‐values of 0.055 for AMVs, 0.162 for RMVs, 0.298 for PMVs, and 0.060 for EMVs (Table 3). However, Spearman correlation analysis revealed a slight positive correlation between age and both AMVs and RMVs (*r* = 0.25), suggesting a modest increase in these MVs subtypes with age. In contrast, PMVs and EMVs showed no meaningful correlation with age (*r* = −0.02 and *r* = −0.10, respectively).

**TABLE 3:** Sex and age‐based comparison of MVs subtypes in SCD patients.

MVs subtype	Male (MVs/μL)	Female (MVs/μL)	*p* (sex)	G1 (< 12 years) (MVs/μL)	G2 (≥ 12 years) (MVs/μL)	*p* (age)	*r*
AMVs^a^	133.6 [48.2–309.9]	62.1 [25.1–180.2]	0.075	150.9 [40.3–325.4]	55.3 [19.8–126.5]	0.055	0.25
RMVs^b^	39.6 [19.7–121.4]	27.6 [7.1–90.5]	0.291	38.8 [10.7–131.0]	27.0 [9.3–50.2]	0.162	0.25
PMVs^c^	32.3 [5.6–163.6]	12.3 [5.6–37.1]	0.123	18.9 [6.1–133.7]	9.9 [2.8–52.2]	0.298	−0.02
EMVs^d^	3.0 [1.2–4.6]	2.4 [0.9–4.2]	0.688	3.1 [1.2–5.2]	1.5 [0.7–2.8]	0.060	−0.10

*Note:* This table summarizes the distribution of MVs subtypes, AMVs, RMVs, PMVs, and EMVs in SCD patients, analysed by sex (male vs. female) and age group (Group 1: < 12 years; Group 2: ≥ 12 years). Mann–Whitney *U* tests showed no statistically significant differences in MV levels between sexes or age groups (all *p* > 0.05). Spearman correlation coefficients (*r*) indicate a weak positive correlation between age and both AMVs and RMVs, and no correlation for PMVs and EMVs. These findings suggest that neither sex nor age has a significant impact on plasmatic MV levels in this cohort.

^a^ Phosphatidylserine‐positive microvesicles.

^b^ Erythrocyte‐derived microvesicles.

^c^ Platelet‐derived microvesicles.

^d^ Endothelial‐derived microvesicles.

### 3.4 Assessment of Plasmatic Microvesicles With Clinical Severity Scores Among SCD Patients

**FIGURE 3:**
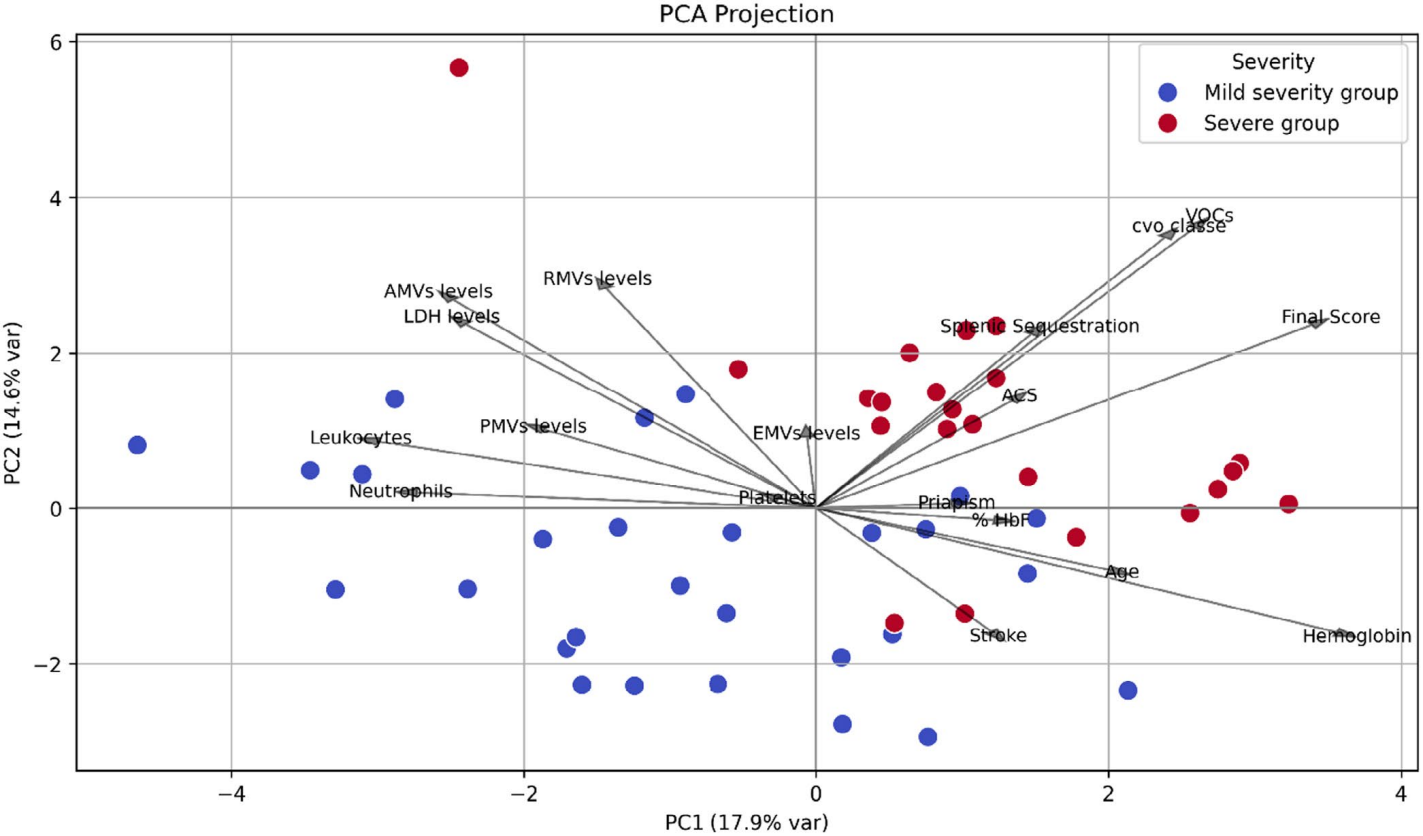
PCA of clinical, biological variables, and plasmatic microvesicles in SCD patients according to severity score. This PCA illustrates the distribution of SCD patients stratified by clinical severity (blue = mild, red = severe). Vectors represent the direction and contribution of variables, including MVs subtypes (AMVs, RMVs, PMVs and EMVs), clinical severity scores (VOC, ACS, splenic sequestration, stroke) and hematological parameters (Hb, Hb F, LDH, leukocytes, neutrophils, platelets). The final severity score vector is closely aligned with AMVs, RMVs and EMVs, indicating a potential association between elevated levels of these MVs and increased clinical severity. In contrast, PMVs appear less strongly associated. Notably, fetal hemoglobin (HbF) is oriented in the opposite direction to the severity score and EMVs, suggesting a potential protective role, whereby higher HbF levels may be associated with reduced EMVs release and milder clinical manifestations.

To assess clinical severity, a scoring system was established based on the presence or absence of specific complications: a score of 3 was assigned for the presence of vaso‐occlusive events (VOC), 3 for splenic sequestration, 3 for acute chest syndrome (ACS), and 5 for stroke (AVC), with a score of 0 assigned if the complication was absent. A cumulative severity score was then calculated for each patient, and individuals were divided into two groups: a mild severity group (final score < 6) and a severe group (final score > 6). The Mann–Whitney *U* test revealed no statistically significant differences in the levels of AMVs, RMVs, PMVs and EMVs between the two groups, with *p*‐values of 0.652, 0.475, 0.464 and 0.796, respectively (Table 4). However, Spearman correlation analysis showed a modest positive correlation between RMVs and the final severity score (*r* = 0.34), while AMVs, PMVs and EMVs showed no correlation. These findings were further supported by PCA (Figure 3), which illustrates the distribution of patients according to clinical severity. The vectors representing AMVs, RMVs, and EMVs are directionally aligned with the final severity score, suggesting that higher levels of these MVs are associated with increased clinical severity. PMVs, in contrast, are oriented differently, indicating a weaker link with severity. The HbF vector points in the opposite direction to the severity score and EMVs, consistent with a potential protective role of HbF in limiting MVs release and reducing clinical complications. This visual representation reinforces the interpretation that specific MVs subtypes may contribute to, or reflect, disease severity in SCD patients.

**TABLE 4:** Association between clinical severity and MV levels in SCD patients.

MVs subtype	Mild severity group: median [IQR] (MVs/μL)	Severe group: median [IQR] (MVs/μL)	*p*	*r*
AMVs^a^	92.690 [28.578–229.900]	69.998 [41.703–296.404]	0.652	0.05
RMVs^b^	27.756 [9.286–110.113]	40.187 [12.561–81.648]	0.475	0.34
PMVs^c^	15.398 [5.217–72.177]	21.577 [6.483–132.298]	0.464	−0.01
EMVs^d^	2.735 [1.174–4.684]	2.735 [0.851–4.305]	0.796	0.03

*Note:* This table presents the comparison of AMVs, RMVs, PMVs, and EMVs levels between patients with mild and severe clinical forms of SCD. The Mann–Whitney *U* test revealed no statistically significant differences between the two groups (*p* > 0.05 for all). However, Spearman correlation analysis revealed a modest positive association between RMVs and the cumulative severity score (*r* = 0.34), whereas other MVs subtypes showed no significant correlation.

^a^ Phosphatidylserine‐positive microvesicles.

^b^ Erythrocyte‐derived microvesicles.

^c^ Platelet‐derived microvesicles.

^d^ Endothelial‐derived microvesicles.

### 3.5 LDH as a Marker of Hemolysis and Its Correlation With MVs Levels

**FIGURE 4:**
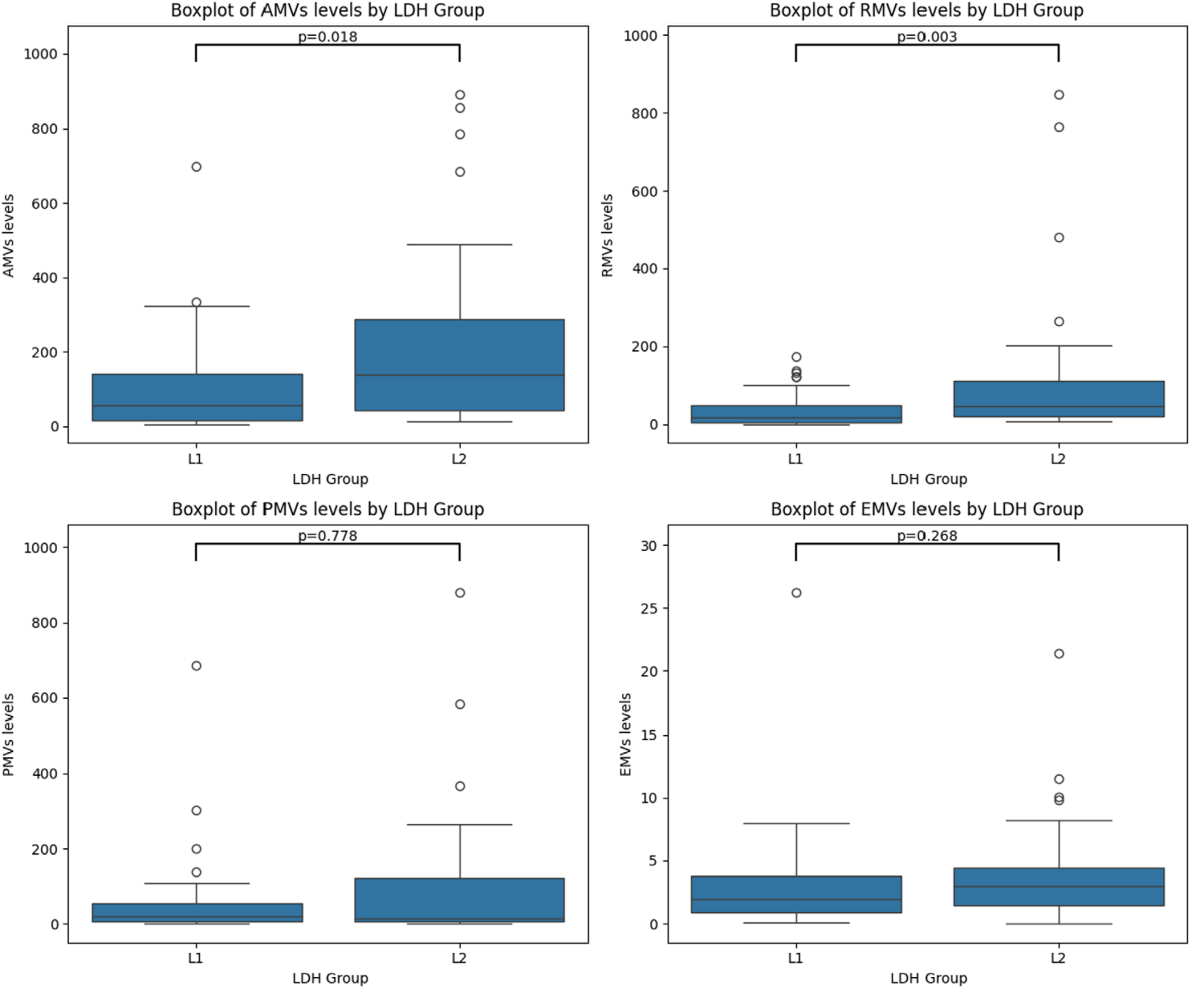
Comparison of MVs subtypes between SCD patients stratified by LDH levels. This figure illustrates the relationship between lactate dehydrogenase (LDH) levels and MVs subtypes in patients with SCD. Patients were divided into two groups: L1 (LDH ≤ 500 IU/L) and L2 (LDH > 500 IU/L). Significantly higher levels of phosphatidylserine‐positive MVs (AMVs; *p* = 0.018) and erythrocyte‐derived MVs (RMVs; *p* = 0.003) were observed in the L2 group, suggesting a potential association between elevated hemolytic activity and increased release of specific MV subtypes. No significant differences were found for platelet‐derived MVs (PMVs; *p* = 0.778) or endothelial‐derived MVs (EMVs; *p* = 0.268).

To further explore the relationship between MVs and hemolysis, patients were stratified according to lactate dehydrogenase (LDH) levels. The analysis showed that patients with LDH > 500 IU/L had significantly higher levels of AMVs (*p* = 0.018) and RMVs (*p* = 0.003)ompared to those with LDH < 500 IU/L. No statistically significant differences were observed for PMVs and EMVs, with *p*‐values of 0.778 and 0.268, respectively (Table 5, Figure 4). PCA indicated that LDH clusters with RMVs and clinical severity. Additionally, Spearman correlation analysis revealed a positive correlation between LDH and RMVs (*r* = 0.32), reinforcing the association between hemolysis and MV release. A strong negative correlation was also found between LDH and Hb (*r* = −0.52), indicating that higher hemolysis is associated with lower Hb levels.

**TABLE 5:** Relationship between LDH and MVs subtypes in SCD patients.

Parameters	Group 1 (LDH < 500 IU/L): median [IQR] (MVs/μL)	Group 2 (LDH > 500 IU/L): median [IQR] (MVs/μL)	*p*	*r*
AMVs^a^	56.424 [14.385–141.465]	137.059 [42.976–287.211]	0.018	0.26
RMVs^b^	17.728 [4.441–48.573]	46.902 [21.425–111.557]	0.003	0.32
PMVs^c^	19.956 [6.534–54.398]	13.828 [4.989–122.345]	0.778	−0.025
EMVs^d^	1.925 [0.881–3.770]	2.955 [1.469–4.458]	0.268	0.14
Hb^e^	9.400 [8.800–10.000]	7.750 [7.175–8.550]	< 0.0001	−0.52

*Note:* This table summarizes the relationship between LDH levels and MVs subtypes in SCD patients. Significantly higher levels of AMVs and RMVs were observed in patients with LDH > 500 IU/L, indicating a potential link between elevated hemolysis and increased MVs release. Spearman correlation analysis confirmed moderate positive correlations of LDH with both AMVs and RMVs. PMVs and EMVs did not show significant differences or meaningful correlations with LDH levels.

^a^ Phosphatidylserine‐positive microvesicles.

^b^ Erythrocyte‐derived microvesicles.

^c^ Platelet‐derived microvesicles.

^d^ Endothelial‐derived microvesicles.

^e^ Hemoglobin.

### 3.6 Effect of Hydroxyurea Treatment on MVs Levels in SCD Patients

To investigate the impact of HU treatment on MVs levels, patients were divided into treated (HU^+^, *n* = 40) and untreated (HU^−^, *n* = 28) groups. The Mann–Whitney *U* test revealed no statistically significant differences in the levels of AMVs, RMVs, PMVs and EMVs between the two groups, with *p*‐values of 0.750, 0.869, 0.924 and 0.343, respectively (Table 6). However, both HU^+^ and HU^−^ groups had significantly higher levels of all MV subtypes compared with healthy controls, suggesting that the increase in MVs release is primarily associated with the disease itself rather than HU treatment.

**TABLE 6:** Comparison of MV levels in HU‐treated, untreated SCD patients and controls.

MVs subtype	Median [IQR] (HU^+^)^e^ (MVs/μL)	Median [IQR] (HU^−^)^f^ (MVs/μL)	*p* (HU^+^ vs. HU^−^)	*p* (controls vs. HU^+^)	*p* (controls vs. HU^−^)
AMVs^a^	86.92 [34.24–290.27]	70.40 [32.92–190.36]	0.750	< 0.0001	< 0.0001
RMVs^b^	32.21 [11.34–91.22]	30.69 [8.44–99.38]	0.869	< 0.0001	0.0003
PMVs^c^	17.82 [5.72–125.66]	15.40 [5.27–85.60]	0.924	0.0002	0.005
EMVs^d^	2.43 [0.81–4.27]	2.84 [1.13–5.17]	0.343	0.0017	0.0005

*Note:* This table presents the levels of MVs subtypes, AMVs, RMVs, PMVs, and EMVs in hydroxyurea (HU)‐treated (HU^+^) and untreated (HU^−^) SCD patients, as well as healthy controls. Mann–Whitney *U* tests revealed no statistically significant differences in any MVs subtype between HU^+^ and HU^−^ patients (all *p* > 0.05), indicating that HU treatment does not significantly influence MVs levels. However, both HU^+^ and HU^−^ patients showed significantly higher levels of all MV subtypes compared to healthy controls (all *p* < 0.01), suggesting a persistent elevation of circulating MVs in SCD independent of HU therapy.

^a^ Phosphatidylserine‐positive microvesicles.

^b^ Erythrocyte‐derived microvesicles.

^c^ Platelet‐derived microvesicles.

^d^ Endothelial‐derived microvesicles.

^e^ Patient with Hydroxyurea.

^f^ Untreated patient.

### 3.7 Association Between Hb F Levels and MVs in SCD Patients

**FIGURE 5:**
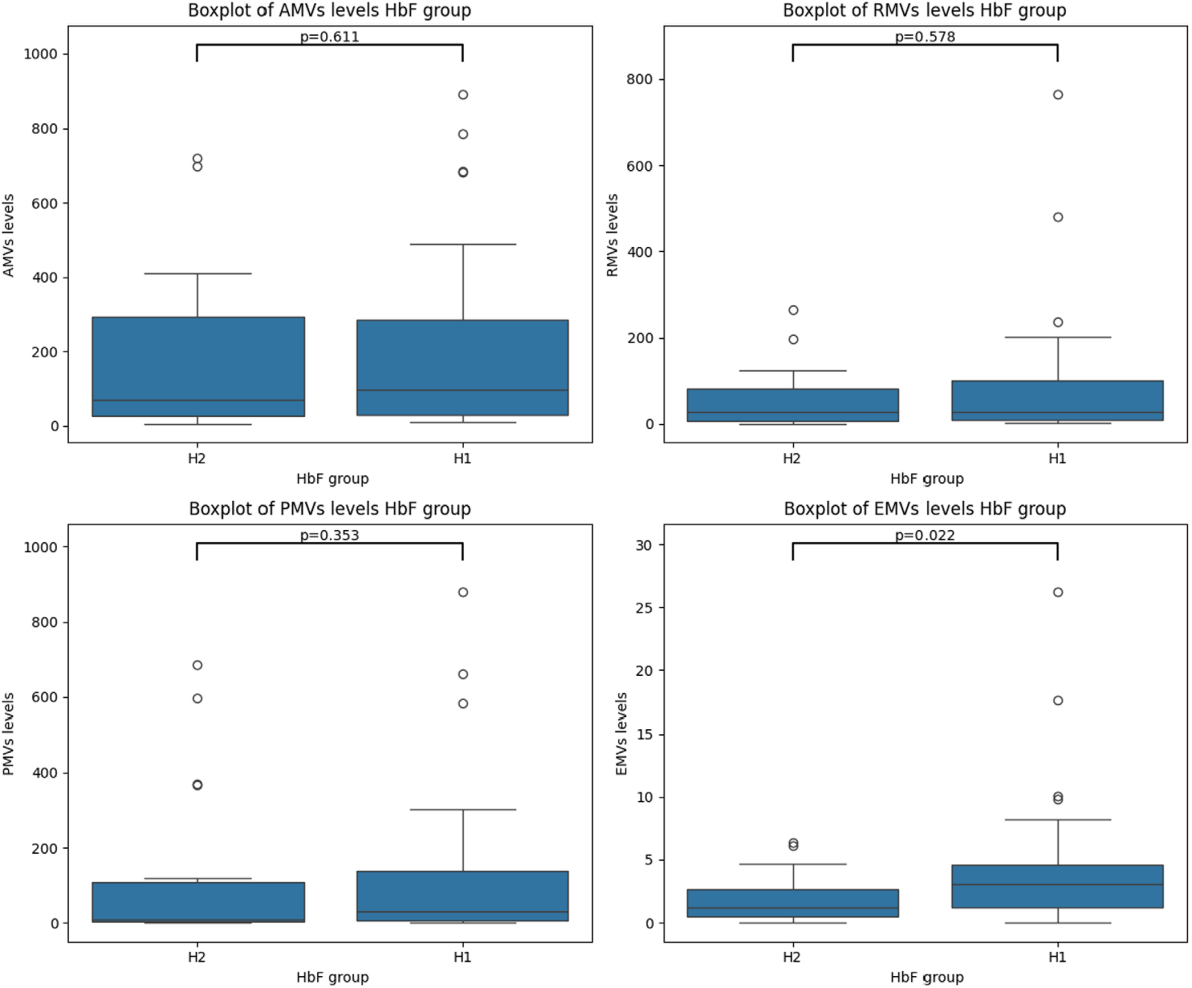
Boxplots of MVs subtypes in SCD patients according to HbF levels. Boxplots illustrate the distribution of MVs subtypes in SCD patients grouped by HbF levels: H1 (HbF < 20%) and H2 (HbF ≥ 20%). No statistically significant differences were observed in the levels of phosphatidylserine‐positive MVs (AMVs; *p* = 0.611), erythrocyte‐derived MVs (RMVs; *p* = 0.578), or platelet‐derived MVs (PMVs; *p* = 0.353) between the two groups. However, endothelial‐derived MVs (EMVs) were significantly lower in the H2 group (*p* = 0.022), suggesting that higher HbF levels may be associated with reduced endothelial activation and a milder disease phenotype.

To understand the role of Hb F, patients were divided into two groups: H1 (Hb F < 20%) and H2 (Hb F ≥ 20%). The Mann–Whitney test revealed no statistically significant differences in the levels of AMVs, RMVs and PMVs between the two groups, with *p*‐values of 0.611, 0.578 and 0.353, respectively. However, a statistically significant difference was observed in EMVs levels between the two groups (*p* = 0.022), with lower EMVs levels in patients with higher Hb F (Table 7, Figure 5), suggesting a potential protective effect of elevated Hb F against endothelial activation or injury. In parallel, PCA projection analysis (Figure 3) showed that higher Hb F levels cluster with less severe clinical phenotypes and are inversely associated with MVs, particularly AMVs and RMVs. In the PCA plot, the Hb F vector points in the opposite direction to EMVs, suggesting that higher Hb F levels may help protect against endothelial damage. This observation is further supported by the Spearman correlation heatmap, which revealed a negative correlation between HbF and EMVs (*r* = −0.27), and a weaker correlation with PMVs (*r* = −0.13). These findings support the hypothesis that Hb F may exert a protective role in SCD, potentially by reducing hemolysis‐induced MVs release and limiting endothelial dysfunction, both of which are critical in the prevention of VOC.

**TABLE 7:** Association between Hb F levels and MVs in SCD patients.

MVs subtype	HbF < 20%: median [IQR] (MVs/μL)	HbF > 20%: median [IQR] (MVs/μL)	*p*	*r*
AMVs^a^	121.560 [41.703–284.147]	62.097 [30.807–211.362]	0.611	−0.063
RMVs^b^	29.681 [9.648–99.375]	39.648 [11.346–91.221]	0.578	0.011
PMVs^c^	37.076 [8.509–163.608]	7.421 [3.090–21.611]	0.353	−0.13
EMVs^d^	2.836 [1.013–5.166]	2.431 [0.917–4.203]	0.022	−0.27

*Note:* No significant differences were observed in the levels of AMVs, RMVs, or PMVs between the low HbF (< 20%) and high HbF (≥ 20%) groups (all *p* > 0.05). However, EMV levels were significantly higher in the low HbF group (*p* = 0.022). This finding is supported by a moderate negative Spearman correlation between HbF and EMVs (*r* = −0.27), suggesting that higher HbF levels may be associated with reduced endothelial activation and lower EMV release.

^a^ Phosphatidylserine‐positive microvesicles.

^b^ Erythrocyte‐derived microvesicles.

^c^ Platelet‐derived microvesicles.

^d^ Endothelial‐derived microvesicles.

## 4 Discussion

The current study compared the levels of MVs between SCD patients and healthy controls and evaluated their variability according to age and sex. It also explored the relationships between MVs levels and biological parameters, specifically Hb, Hb F and LDH, as well as clinical severity scores.

Our findings revealed significantly higher levels of AMVs, RMVs, PMVs and EMVs in SCD patients compared to healthy donors. Specifically, median concentrations in our cohort were 78.66, 31.45, 16.31 and 2.74 MVs/μL for AMVs, RMVs, PMVs and EMVs, respectively, compared with 18.03, 4.88, 6.03 and 0.87 MVs/μL in the control group. These results, which are specific to the Tunisian population, are in line with previous studies reporting increased MVs levels in SCD, primarily originating from erythrocytes and platelets, followed by leukocytes and endothelial cells. Nebor et al. observed a 3‐ to 4‐fold increase in total MVs in SCD patients compared to controls (4149 [857–26,863] vs. 1410 [696–7158] MVs/μL), with RBC‐ and platelet‐derived MVs being the most frequently detected [31]. Similarly, Nader et al. reported elevated medium/large MVs, primarily originating from RBCs and platelets, with lower levels from other blood cell types. Their study also noted that the concentration and density of externalised phosphatidylserine on these MVs may vary according to clinical status and treatment [13]. Minor differences in absolute concentrations between studies may reflect variations in flow cytometry protocols, sample preparation, or patient populations. Overall, the elevation in MVs reflects enhanced cell activation, apoptosis, and endothelial dysfunction, processes closely linked to hemolysis and thrombotic risk [13, 31, 32].

Sex and age did not appear to significantly influence MVs levels in our cohort. No significant differences were observed between males and females, which is consistent with findings from other paediatric SCD studies [33, 34]. However, some studies in adult populations suggest sex‐related differences in disease complications, often attributed to hormonal changes after puberty. While males may experience more severe clinical complications, these differences do not seem to directly impact MVs levels in children [35].

Similarly, age did not significantly affect MVs profiles. No differences were found between the < 12 and > 12 years age groups. However, Spearman correlation analysis showed a modest positive correlation between age and both AMVs and RMVs, suggesting a slight age‐related increase in these MVs subtypes. In contrast, PMVs and EMVs did not show any meaningful correlation with age. These findings differ from some previous studies that reported a clear age‐related increase in circulating MVs levels in children with SCD, where younger children exhibited significantly lower total MVs concentrations compared to older ones [36].

Clinical severity was determined using a composite score based on complications such as VOC, ACS, splenic sequestration and stroke. The comparison between the mild and severe groups revealed no statistically significant differences in MVs levels. However, correlation analysis showed a modest positive association between RMVs levels and the final clinical severity score, a finding further supported by principal component analysis (PCA). These observations suggest a potential link between MVs levels, particularly RMVs, and disease severity in SCD patients.

This association may reflect the pathophysiological role of RMVs in SCD, as they are released from erythrocytes undergoing eryptosis and hemolysis. RMVs may also be generated by mechanical stress during passage through the spleen, where cyclic forces alter erythrocyte membrane composition and trigger vesiculation [37]. Elevated RMVs levels indicate increased membrane stress and oxidative damage, which can contribute to endothelial injury and an elevated thrombotic risk.

Several studies have highlighted associations between MV levels and SCD complications. For example, high PMV levels have been linked to frequent VOC episodes, while elevated RMV levels are primarily associated with increased haemolysis [31]. Other studies reported increased AMV levels during acute crises [38], and both RMV and PMV were found to be significantly elevated in splenectomised patients [39].

LDH is a common biochemical marker of intravascular hemolysis in SCD. In our study, significantly higher levels of AMVs and RMVs were observed in patients with LDH values exceeding 500 IU/L, suggesting a potential association between elevated hemolytic activity and the increased release of MVs, particularly RMVs. This finding was supported by PCA, which demonstrated that LDH clustered with RMVs and clinical severity, and by Spearman correlation analysis, which revealed a positive correlation between LDH and RMVs, and a negative correlation between LDH and Hb levels.

These results reinforce the hypothesis that increased hemolysis contributes to RMVs release in SCD. Several studies are consistent with our findings, reporting a positive correlation between elevated LDH levels and RMVs release [40, 41]. Others have also highlighted the association between elevated LDH and RMVs levels with increased coagulation activation and endothelial dysfunction in SCD [12, 42]. In conclusion, both RMVs and LDH may serve as key biomarkers of hemolysis severity in SCD and appear to contribute to the disease's pathophysiology by promoting vascular injury and prothrombotic states.

In SCD, hydroxyurea (HU) is the most commonly used standard therapeutic approach. It increases HbF production, reduces haemolysis, and improves clinical outcomes [43]. HU has also been reported to decrease coagulation activation markers, such as thrombin–antithrombin complexes and D‐dimers [44] and to reduce leukocyte and platelet counts while lowering endothelial adhesion molecule expression [45].

In our study, MVs levels did not differ significantly between HU‐treated and untreated patients, suggesting that HU does not substantially influence MVs release. Nevertheless, both HU^+^ and HU^−^ groups showed elevated MVs levels compared with healthy controls. Previous studies have reported higher total MVs concentrations, particularly those derived from erythrocytes, monocytes, and endothelial cells, in HU‐treated patients relative to untreated ones. These increases have been hypothesized to result from HU‐induced changes in cell membrane properties during megaloblastic processes [44]. We did not directly investigate these mechanisms in our study.

Hb F is considered a major modulator of SCD severity primarily by inhibiting the polymerization of Hb S and thereby reducing hemolysis and cellular activation [46]. In the present study, MV levels were evaluated according to Hb F levels, dividing patients into two groups: Hb F < 20% and Hb F ≥ 20%. The analysis revealed a statistically significant difference in EMV levels between the two groups, with decreased EMV concentrations observed in patients with higher Hb F levels. This result was supported by Spearman correlation analysis, which demonstrated a negative correlation between Hb F and EMV. Furthermore, PCA analysis showed that higher Hb F levels clustered with milder clinical phenotypes and were inversely associated with MV, particularly EMV. These findings suggest that elevated Hb F may contribute to protecting against endothelial damage and reducing endothelial activation. Other studies have similarly reported negative correlations between Hb F and absolute MV concentrations derived from erythrocytes, platelets, and monocytes [36, 47]. Hb F appears to play a protective effect in SCD not only by stabilising red blood cells and preventing sickling and hemolysis but also by limiting the release of procoagulant and proinflammatory MV from various blood cell types. Consequently, higher Hb F levels may reduce endothelial injury and the risk of vascular complications in SCD.

Overall, our findings highlight the potential role of MVs as markers of cellular stress, haemolysis, and vascular dysfunction in SCD. The significant associations between MVs, particularly RMVs and EMVs, and clinical and biological parameters such as LDH, Hb F, and disease severity suggest that MVs may serve as valuable biomarkers for SCD diagnosis and for identifying patients at risk of complications. Given their sensitivity to haemolytic and endothelial activity, MVs could be promising tools for both diagnostic and prognostic use. Further research is essential to confirm these findings and explore their potential as therapeutic targets.

## Author Contributions

**Khouloud Khalfaoui:** formal analysis (equal), investigation (equal), methodology (equal), visualization (equal), writing – original draft (lead). **Mariem Chebbi:** investigation (equal), methodology (equal). **Oussema Souiai:** formal analysis (equal), visualization (equal). **Ilhem Ben Fraj:** investigation (supporting). **Monia Ben Khaled:** investigation (supporting). **Amine Hableni:** formal analysis (supporting). **Mbarka Barmate:** methodology (supporting). **Dorra Chaouachi:** methodology (supporting). **Fethi Mellouli:** investigation (supporting). **Monia Ouederni:** investigation (supporting). **Ines Safra:** conceptualization (equal), supervision (equal), writing – review and editing (equal). **Samia Menif:** project administration (supporting), supervision (supporting). **Imen Moumni:** conceptualization (equal), project administration (lead), supervision (equal), writing – review and editing (equal).

## Ethics Statement

The study was conducted in accordance with the Declaration of Helsinki and was approved by the Institutional Ethics Committee of the Institut Pasteur de Tunis (protocol code 2018/11/I/LR16IPT07/V1).

## Consent

Informed consent was obtained from all participants involved in this study. Written informed consent has been obtained from the participants to publish this paper.

## Conflicts of Interest

The authors declare no conflicts of interest.

## Data Availability

The data that support the findings of this study are available from the corresponding author upon reasonable request.
